# Malaria vaccine efficacy, safety, and community perception in Africa: a scoping review of recent empirical studies

**DOI:** 10.1007/s15010-024-02196-y

**Published:** 2024-03-05

**Authors:** Muhammad Chutiyami, Priya Saravanakumar, Umar Muhammad Bello, Dauda Salihu, Khadijat Adeleye, Mustapha Adam Kolo, Kabiru Kasamu Dawa, Dathini Hamina, Pratibha Bhandari, Surajo Kamilu Sulaiman, Jenny Sim

**Affiliations:** 1 School of Nursing and Midwifery, University of Technology Sydney, Sydney, Australia; 2 Department of Physiotherapy and Paramedicine, School of Health and Life Sciences, Glasgow Caledonian University, Glasgow, UK; 3 College of Nursing, Jouf University, Sakaka, Saudi Arabia; 4 College of Nursing, University of Massachusetts, Amherst, MA 01003 USA; 5 Department of Geography, University of Maiduguri, Maiduguri, Nigeria; 6 School of Nursing, Midwifery and Health Education, University of Bedfordshire, Luton, UK; 7 Department of Nursing Science, University of Maiduguri, Maiduguri, Nigeria; 8 Department of Physiotherapy, Tishk International University, Erbil, Iraq; 9 WHO Collaborating Centre for Nursing, Midwifery and Health Development, University of Technology Sydney, Sydney, Australia; 10 School of Nursing, Midwifery and Paramedicine, Australian Catholic University, Sydney, Australia

**Keywords:** Malaria vaccine, Efficacy, Safety, Perception, Africa

## Abstract

**Aim:**

The review summarizes the recent empirical evidence on the efficacy, safety, and community perception of malaria vaccines in Africa.

**Methods:**

Academic Search Complete, African Journals Online, CINAHL, Medline, PsychInfo, and two gray literature sources were searched in January 2023, and updated in June 2023. Relevant studies published from 2012 were included. Studies were screened, appraised, and synthesized in line with the review aim. Statistical results are presented as 95% Confidence Intervals and proportions/percentages.

**Results:**

Sixty-six (*N* = 66) studies met the inclusion criteria. Of the vaccines identified, overall efficacy at 12 months was highest for the R21 vaccine (*N* = 3) at 77.0%, compared to the RTS,S vaccine (*N* = 15) at 55%. The efficacy of other vaccines was BK-SE36 (11.0–50.0%, *N* = 1), ChAd63/MVA ME-TRAP (− 4.7–19.4%, *N* = 2), FMP2.1/AS02A (7.6–9.9%, *N* = 1), GMZ2 (0.6–60.0%, *N* = 5), PfPZ (20.0–100.0%, *N* = 5), and PfSPZ-CVac (24.8–33.6%, *N* = 1). Injection site pain and fever were the most common adverse events (*N* = 26), while febrile convulsion (*N* = 8) was the most reported, vaccine-related Serious Adverse Event. Mixed perceptions of malaria vaccines were found in African communities (*N* = 17); awareness was generally low, ranging from 11% in Tanzania to 60% in Nigeria (*N* = 9), compared to willingness to accept the vaccines, which varied from 32.3% in Ethiopia to 96% in Sierra Leone (*N* = 15). Other issues include availability, logistics, and misconceptions.

**Conclusion:**

Malaria vaccines protect against malaria infection in varying degrees, with severe side effects rarely occurring. Further research is required to improve vaccine efficacy and community involvement is needed to ensure successful widespread use in African communities.

**Supplementary Information:**

The online version contains supplementary material available at 10.1007/s15010-024-02196-y.

## Introduction

Malaria is prevalent in Africa and poses a significant public health threat with substantial morbidity and mortality [1]. Despite concerted efforts to curb the disease, its persistence can be attributed to socioeconomic inequality, inadequate infrastructure, and the emergence and spread of drug-resistant strains [2]. Control measures such as insecticide-treated nets (ITNs), indoor residual spraying (IRS), and antimalarial drugs are critical, but additional complementary interventions are needed. One of the promising emergent strategies is vaccination, which has been identified as a potentially pivotal measure in the fight against malaria [3].

Developing a malaria vaccine has been an arduous journey, complicated by the inherent complexity of the Plasmodium parasite's life cycle and its diverse antigenic characteristics [4]. Despite these challenges, there has been substantial progress. One particular advancement in this field is the RTS,S/AS01 and the R21/Matrix-M vaccines. These vaccines demonstrated protective efficacy in large-scale clinical trials, and have been recommended by the World Health Organization (WHO) for use in regions with moderate to high *P. falciparum* transmission, particularly Sub-Saharan Africa [5].

Malaria vaccine clinical trials have provided important knowledge and insights to support the implementation of large-scale vaccination programs. Mokuolo et al. [6] offered several key learnings from these trials, stressing the significance of robust local regulatory and ethical frameworks, effective community engagement and communication, as well as vigilant monitoring for potential disease enhancement or rebound morbidity following temporary interruptions of clinical infections. A critical factor in the success of vaccine implementation is community acceptance. A recent review of the literature suggests high acceptance of the RTS,S malaria vaccine across low- and middle-income countries (LMICs), with an average acceptance rate of 95.3% [7]. However, acceptance rates vary and appear to be impacted by socio-demographic factors and community apprehensions about safety, efficacy, and vaccine awareness [8, 9].

In light of the success of the RTS,S and R21 vaccines, the need for greater global resources for malaria vaccine research and logistics in vaccine implementation cannot be over-emphasized. This study sought to address a current gap in understanding by using an in-depth scoping review to summarize recent empirical evidence on malaria vaccine efficacy, safety, and community perceptions in Africa.

## Methods

A scoping review was conducted using the methodological framework outlined by Arksey and O’Malley [10], incorporated quality recomendations [11], and reported using the PRISMA extension for scoping reviews (PRISMA-ScR), as outlined in Appendix 1 [12]. The review protocol was registered at Open Science Framework (OSF) at https://doi.org/10.17605/OSF.IO/D54YC.

### Eligibility criteria

Studies were included if they evaluated the efficacy, safety, or community perception of a malaria vaccine; were published after 2011; were primary/empirical research; conducted in malaria-endemic African countries; and included the general public as participants (e.g., caregivers, parents, children, or adults). Studies published from 2012 were included as a previous review that have explored malaria vaccine research prior to 2012 [13]. Studies were excluded if the participants were outside Africa, were not primary research (reviews, opinions, editorial, commentaries), and if they evaluated immunogenicity without safety or efficacy as a construct.

### Information sources

Five primary databases were searched to identify relevant studies in any language: African Journals Online (AJOL), Academic Search Complete, Medline, CINAHL and PsychInfo. The initial search was conducted in January 2023 for articles published from 2012 to 2022. An update search was conducted in June 2023 for articles published from 2022 to June 2023. The search was supplemented with two gray literature sources; AfricArxiv (Achieve for African Research) and OPUS (Open Publication of UTS Scholars) to identify relevant preprints and thesis/dissertations respectively. Additionally, the reference list of articles that met the inclusion criteria was searched manually and forward literature search on Google Scholar was conducted to identify potentially missing articles. Peer review identified three additional studies published after June 2023 and those studies have also been included.

### Search

A combination of MeSh and index terms were formulated based on the PICO framework to aid the search process: Population (P)—African communities, Intervention (I)—malaria vaccine, Comparator (C)—none, and Outcome (O)—efficacy, safety, community perception. The EBSCOhost interface (including Academic Search Complete, CINAHL, Medline with full-text and PsychInfo) and the AJOL database were searched. The full search terms are reported in supplemental Table S1. The EBSCOhost interface was expanded to; ‘Apply related words’ and ‘Apply equivalent subjects’.

For gray literature sources, the term 'malaria vaccine' was used to search for preprints papers on AfricArxiv, and any relevant thesis/publication on OPUS.

### Selection of studies

Two reviewers (MC and KA) screened potentially eligible studies using the eligibility criteria. First, exact duplicates were removed in EBSCOhost and the search was narrowed to studies published from January 2012. Search results were then exported to Endnote. The duplicate screening was conducted in Endnote. The remaining articles were independently screened by 2 reviewers based on the title and abstract. The full text of all potentially relevant articles was then retrieved and screened independently by MC and UMB in-line with the eligibility criteria.

### Data charting process

A data extraction form was developed by three authors (MC, UMB, DS) and included study characteristics such as the citation, year of publication, study design, and study setting. Data related to the study findings varied based on the focus of the study and included the study methods, the type of malaria vaccine assessed, the outcome assessments used, and the major findings. Two reviewers (KA and MAK) independently conducted the data extraction. Differences were resolved through discussion between the two reviewers and a third reviewer (MC).

### Critical appraisal of included studies

The quality of the included studies was assessed using Joanna Briggs Institute (JBI) appraisal tools [14] and the Mixed Methods Appraisal Tool (MMAT) [15]. The appraisal was conducted independently by 2 reviewers (KKD and PB) and differences were resolved by a third reviewer (UMB). No study was excluded based on quality appraisal, but the quality of the study was considered when reaching key conclusions. JBI and MMAT do not provide a scoring guideline, therefore, studies were considered ‘above-average quality’ when they met at least half (average) of the quality criteria assessed in the specific study design. Therefore, the terms ‘below-average quality’ or ‘above-average quality’ were used to refer to study quality in the results.

### Data items

Efficacy was operationally defined as the vaccine’s estimated effect on all malaria episodes (clinical, severe, or hospitalization). Efficacy was based on Intention-To-Treat (ITT) or According-To-Protocol/Per Protocol (ATP) analyses. Where ITT and ATP analyses were unavailable, efficacy was based on Hazard Ratio (HR), or any other percentage/proportion estimates reported in the studies. Safety was defined based on the presence or absence of Adverse Event (AE) and/or Serious Adverse Event (SAE). Community perception was defined as the different views of communities (general population) about malaria vaccines.

### Synthesis of results

Results were synthesized narratively by summarizing the descriptive numerical data followed by a summary of the textual data. The synthesis considered the nature of the research (e.g., design), the type of malaria vaccine (for efficacy and safety), and the quality of the research studies.

Overall efficacy was classified as positive, none/negative or mixed. A result was considered as having positive efficacy if the Confidence Intervals (CI) were within the positive range; mixed efficacy if the CI ranged from negative to positive; and negative efficacy if the CI was within the negative range to zero. Similarly, safety issues were classified based on the number of subjects presenting with at least one SAE, AE, or none. Where the number of affected subjects were not available, a total number of events/incidents was reported. AEs can be solicited, unsolicited or unexpected, and the cumulative number/range was reported based on available information. For community perception, results were synthesized thematically by reporting the overall quantitative results followed by a summary of qualitative results as applicable. Overall percentages/proportions were reported with a range when available. Community perception was further classified based on 3 components: nature of the vaccine (e.g., risks, effect), systems (e.g., mistrust, logistics), or personal reasons (encompassing anything else). *N* refers to the number of studies reporting the same finding, while *n* refers to the number of participants reporting a finding in a study in this review.

## Results

**Fig. 1:**
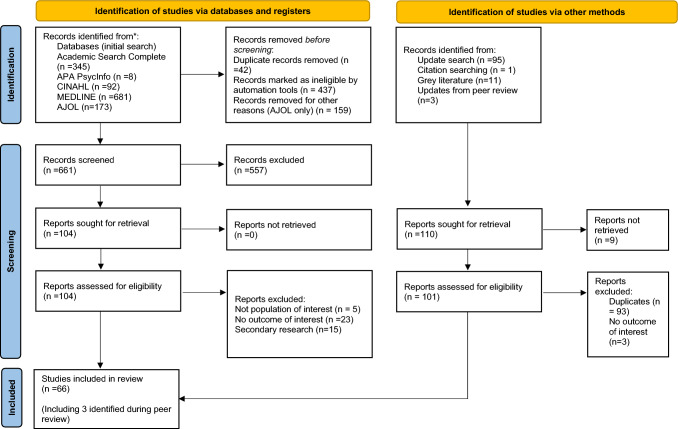
PRISMA flow diagram indicating screening process

We initially found 1299 articles (Fig. 1) from the five databases, and 661 underwent title/abstract screening. Two non-English articles, in Danish and French, were evaluated and excluded as they were secondary research. In total, 66 studies (*N*) were included (61 from the main search, 2 from the updated search, and 3 were identified during peer review) [16–78].

### Characteristics of included studies

The 66 included studies incorporated 47 Randomized Controlled Trials/clinical trials (71.4%), a case–control study (1.6%), and 17 surveys (27.0%). Sixteen African countries were included, with 64 of the 66 studies (97.0%) being above-average quality (Table S2). Further details are presented in Table S3.

### Efficacy of malaria vaccines

Half of the included studies (50%, *N* = 33) reported vaccine efficacy. At 12 months post-vaccination, the R21 vaccine showed the highest overall efficacy at 77% (*N* = 1, *n* = 146), compared to the RTS,S vaccine at 55% (*N* = 1, *n *= 273). Both of these studies were of above-average quality (Table S2). R21 further demonstrated an efficacy of 79% among younger children (5–17 months compared to 18–36 month-olds) at 12 months [86] and 80% (*N* = 1, *n* = 137) at 12 months after a booster dose [78]. Similarly, RTS,S vaccine showed an efficacy of 56% among children aged 5–17 months at 12 months following vaccination [62]. PfSPZ, though tested on only five individuals, demonstrated an efficacy of 100% at three- or eleven-weeks post-vaccination. This efficacy rose from 20 to 100% at 3 weeks when PfSPZ's dosage regimen was adjusted [39]. The combined use of RTS,S/AS01 with chemoprevention yielded efficacy between 59.6 to 60.1% against clinical malaria and outperformed the vaccine in isolation against severe malaria and related deaths [25]. Other vaccines' efficacies varied significantly (Table 1).

**Table 1:** Efficacy of malaria vaccine

Malaria vaccine	Dose	References	Design	Effect			Comments
				Positive	Mixed	Negative	
RTS,S (RTS,S/AS01, RTS,S/AS02)	3 doses	Abdulla et al. [16]	RCT		✔		Efficacy among 50.7% (95% CI 6.5–77.1) after 12 months and 26.7% (95% CI − 33.1 ∓ 59.6) after 18 months post-vaccination
	3 doses	Ajua et al. [19]	RCT	✔			Malaria risk reduction; 54% (HR = 0.46; 95% CI 0.22–0.99) based on quality/avidity, and 77% (HR = 0.23, 95% CI 0.11–0.51) in quantity of IgG concentration
	3 doses	Bejon et al. [22]	RCT		✔		Efficacy changed from 36% (95% CI 24–45) at time of vaccination to 0% (95%CI − 38 ∓ 8) after 3 years
	3 doses + 2 boosters	Cairns et al. [24]	RCT	✔			Efficacy of vaccine among SMC recipients 60.1% (95% CI 52.9–66.2) in the first 6 months and 35.9% (95% CI 0.8–58.6) between 6- and 12 months post-dose five
	3 doses + 2 boosters	Chandramohan et al. [25]	RCT		✔		Vaccine alone efficacy compared to chemoprevention: 7.9% (95%CI − 1.0 ∓ 16) clinical malaria. Efficacy of combination (vaccine + chemo) as compared with chemoprevention alone: 62.8% (95% CI 58.4–66.8) for clinical malaria; 70.5% (95% CI 41.9–85.0) for hospital admission with severe malaria; 72.9% (95% CI 2.9–92.4) for death from malaria. Efficacy of the combination as compared with the vaccine alone: 59.6% (95% CI 54.7–64.0) clinical malaria, 70.6% (95% CI 42.3–85.0) severe malaria; 75.3% (95% CI 12.5–93.0) deaths
	3 doses	Dobano et al.[32]	Case–control	✔			Protection against clinical malaria OR ranges from 0.52 (95%CI 0.26–0.98) to 0.53 (95%CI 0.3–0.93)
	3–4 doses	Tinto et al.[73]	RCT		✔		Efficacy against severe malaria; 3-5 years group- 4dose 32·1% (95%CI − 53·1 ∓ 69·9); 3dose group 37·6% (95%CI − 44·4 ∓ 73·0). 5–7 years–dose 53·7% (95% CI − 13·7 ∓ 81.1); 3dose 23·3% (95% CI − 67.1 ∓ 64·8)
	3 doses	Neafsey et al.[49]	RCT	✔			Efficacy of 50.3% (95% CI 34.6–62.3) (HR: 62.7%) when parasites matched the vaccine compared with 33.4% (95%CI 29.3–37.2) (HR: 54.2%) against mismatched malaria after 1 year
	3 doses	Olotu et al.[54]	RCT		✔		Efficacy was 43.6% (95% CI 15.5–62.3) in the first year but was − 0.4% (95% CI – 32.1 ∓ 45.3) in the 4th year
	3 doses	Olotu et al.[55]	RCT		✔		7 year follow-up; ITT analysis overall 4.4% (95%CI − 17.0 ∓ 21.9); 5 year follow-up in high exposure children − 43.5% (95%CI − 100.3–− 2.8)
	3 doses	RTS,S Clinical Trials Partnership[61]	RCT		✔		Efficacy (ITT) of 30.1% (95% CI 23.6–36.1); efficacy against severe malaria was 26.0% (95% CI − 7.4 ∓ 48.6)
	3 doses	RTS,S Clinical Trials Partnership[62]	RCT		✔		Clinical malaria (ITT): 5–17 months 45% (95% CI 41.0–49.0); 6–12 weeks 27% (95% CI 21.0–33.0). Severe malaria (ITT): 5–17 months 34% (95% CI 15.0–48.0); 6–12 weeks 8% (95% CI − 26.0 ∓ 33.0)
	3–4 doses (4th dose booster)	RTS,S Clinical Trials Partnership[63]	RCT		✔		Clinical malaria: 5–17 months 36·3% 4 doses, (95% CI 31·8–40·5), 28·3% 3 doses, (95% CI 23·3–32·9); 6–12 weeks 25·9% 4 doses, (95% CI 19·9–31·5), 18·3% 3 doses, (95% CI 11·7–24·4). Severe malaria: 5–17 months 4 doses, 32·2%, (95% CI 13·7–46·9), 1·1% 3 doses, (95% CI − 23·0 ∓ 20·5); 6-12 weeks 4 doses, 17·3% (95% CI − 9·4 ∓ 37·5), 10·3% 3 doses, (95CI − 17·9–31·8)
	3 doses + 2 boosters	Sagara et al.[65]	RCT	✔			Protective efficacy against clinical malaria based on below and above antibody threshold: 40.4% (95%CI 16.0–57.7) post-dose 5
	Variable doses 1–4 doses	Samuels et al.[66]	RCT		✔		Over 12 months, Efficacy against clinical malaria (all episodes): ranged from 35% (95%CI 17.0–49.0) to 55% (95%CI 41.0–66.0); incremental vaccine efficacy in fractional dose group versus pooled full dose and standard regimen groups was − 21% (95% CI − 57.0 ∓ 7.0)
BK-SE36	2 doses	Palacpac et al.[59]	RCT		✔		Projective efficacy of 11% (95%CI − 42.0 + 44.0) > 500parasites/µL and 50% (95%CI 9.0–73.0) parasite density > 5000 parasites/L and any axillary temperature
ChAd63 ME-TRAP followed by MVA ME-TRAP	2 doses	Mensah et al.[46]	RCT		✔		Efficacy 8%for any PCR positive (95% CI − 100.0 ∓ 59.0); 9% for > 10parasites/ml (95%CI − 180.0 ∓ 50.0)
	2 doses	Tiono et al.[74]	RCT		✔		Efficacy after 6 months; 13.8% (95%CI − 42.4 ∓ 47.9) uncomplicated malaria; 19.4% (95%CI − 58.9− + 59.1) and − 4.7% (95%CI − 114.0 ∓ 48.8) in unadjusted and adjusted cohorts for severe malaria
FMP2.1/AS02A	3 doses	Laurens et al. [42]	RCT		✔		Efficacy was 7.6% (95%CI − 16.7 ∓ 26.8) against first clinical malaria episodes and 9.9% (95%CI − 5.4 ∓ 23.0%) against all malaria episodes within 0–24 months
GMZ2 (GMZ2-CAF01, GMZ2-alum)	3 doses	Dassah et al. [29]	RCT		✔		Overall efficacy of 6.5% (95%: CI − 1.6 ∓ 14.0) ITT for 2 years. Year 1: 11.7% (95%CI 3.1–19.6); Years 2 0.6% (95%CI − 10.2 ∓ 10.2)
	3 doses	Dejon-Agobe et al. [31]	RCT	✔			Estimated based on CHMI, resulting in 15/34 (44%) with malaria (parasitemia and symptoms). Vaccine-specific HR: 0.10 (95%CI 0.01–0.9)
	3 doses	Nouatin et al. [50]	RCT		✔		Estimated based on CHMI. HR based on risk of infection using HLA-G concentration: 2.50 (95%CI 1.0–6.0). Proportions/percentages unclear
	3 doses	Nouatin et al. [51]	RCT	✔			Estimated based on CHMI. Efficacy in treatment arm ranged from 25% (2/8) to 60% (6/10)
	3 doses	Sirima et al. [67]	RCT	✔			Overall adjusted efficacy 14% (95%CI 3.6–23.0) per protocol and 11.3% (95%CI 2.5–19.0) in ITT.VE severe malaria 27% (95%CI 44.0–63.0)
PfSPZ	5 doses	Jongo et al. [37]	RCT	✔			Efficacy 20% (95%CI: 4.6–35.4) at 3 weeks post-vaccination
	3 doses	Jongo et al. [39]	RCT	✔			Increasing dose from 2.7 × 10^5^ to 9 × 10^5^ PfSPZ increased VE from 20 to 100% (95%CI 38.3–100.0) at 3 or 11 weeks, but increasing to 1.8 × 10^6^ significantly reduced VE to 33% (95%CI 9.30–70.40) at 7.5 weeks
	3 doses	Oneko et al.[57]	RCT		✔		Efficacy of 45.8%, (95%CI 6.9–68.5) after 3 months, but no significant effect from 6 to 12 months
	5 doses	Sissoko et al. [68]	RCT	✔			Efficacy (1-HR) 0.517 (95%CI 0.3–0.9) by time-to-infection analysis (i.e., efficacy = 52%), and 0.712 (95%CI 0.5–0.9) by proportional analysis
	3 doses	Sissoko et al. [68]	RCT	✔			Efficacy (1-HR) 0·51 (95% CI 0·20–0·70.0) time to infection analysis (efficacy = 51%) and 0.39 (95%CI 0.04–0.6) by ITT; efficacy (1-RR) 0·24 per-protocol (95%CI 0·02–0·4) and 0·22 (95%CI 0·01–0·39) by ITT
PfSPZ-CVac	3 doses	Coulibaly et al. [27]	RCT		✔		Efficacy of 33.6% by HR (95%CI − 27.9 ∓ 65.5) and 24.8% by RR (95%CI − 4.8 ∓ 54.3)
R21	3 doses	Datoo et al. [30]	RCT	✔			Efficacy of 71.0% (95% CI 59.0–79.0) among low-dose and 77% (95%CI 67.0–84.0) among high-dose groups after 1 year
	3 doses + 1 booster	Datoo et al. [78]	RCT	✔			Efficacy of 71.0% (95%CI 60.0–78.0) among low-dose and 80% (95%CI 72.0–85.0) among high-dose groups after 2 years
	3 doses + 1 booster	^a^Datoo et al. [86]	RCT	✔			Efficacy of 72.0% (95%CI 69.0–75.0), ranged from 67% (95%CI 59–73) in standard site to 75% (95%CI 71.0–78.0) in seasonal site against multiple clinical malaria episodes

Abbreviations and Notations—BK-SE36: Plasmodium falciparum serine repeat antigen-5 formulated with aluminium hydroxyl gel; ChAd63: Chimpanzee Adenovirus 63; CHMI: Controlled Human Malaria Infection; CT: Clinical Trial; FMP2.1/AS02A: Plasmodium falciparum apical membrane antigen 1; GMZ2: 2 *Plasmodium falciparum* antigens glutamate-rich protein and merozoite surface protein 3; HLA-G: Human Leukocyte Antigen G; HR: Hazard Ratio; ITT: Intention-to-treat; MSP: *Merozoite surface protein;* MVA: Modified Vaccinia Ankara; ME-TRAP: Multiple Epitope thrombospondin-related adhesion protein; OR: Odds Ratio; *PfAMA1-FVO* Recombinant protein *Pichia pastoris*- expressed apical membrane antigen-1 from *Plasmodium falciparum* FVO clone adsorbed to Alhydrogel; Pfs25H-EPA: Recombinant pichia-expressed, His-tagged-Pfs25-conjugated to an *Escherichia coli*-expressed recombinant; *SMC* seasonal malaria chemoprevention; *EPA* *Pseudomonas aeruginosa* ExoProtein A; *PfSPZ* *Plasmodium falciparum* sporozoite; *PfSPZ-CVac* *Plasmodium* *falciparum* sporozoite chemoprophylaxis vaccine, *R21* Matrix-M/circumsporozoite protein-based Vaccine, *RCT* randomized controlled trial, *RR* risk ratio *RTS,S* recombinant protein malaria vaccine, *VE* vaccine efficacy

^a^Article identified during peer-review

Two studies [55, 73] evaluated the long-term (up to 7 years) efficacy of RTS,S on severe and clinical malaria. While the study by Tinto et al. [73] demonstrated a decrease in severe malaria cases over time, there was a rebound against clinical malaria among older children (5–7 years). Oluto et al. [55] identified that vaccine efficacy (clinical malaria) waned over time, including negative efficacy among children with higher exposure to the malaria parasite. Similarly, a negative efficacy of ChAd63/MVA ME-TRAP for an adjusted severe malaria cohort was found [74]. Vaccine effectiveness was maintained when co-administered with malaria chemoprevention [24, 25, 27] or other childhood vaccinations [20].

### Safety of malaria vaccines

Thirty-six studies (54.5%, *N* = 36) investigated the safety of the malaria vaccines, all employing Randomized Controlled Trial design with above-average quality (Table S2). Each study reported one or more AEs (*N* = 28) or SAEs (*N* = 23). The reported AEs and SAEs ranged broadly across various vaccines; RTS,S (AEs: 1.6–87.5%, *N* = 6; SAEs: 2.8–92.2%, *N* = 12, vaccine-related SAEs: 0.1–1%, *N* = 7), BK-SE36 (AEs: 5.6–94.4%, *N* = 1; SAEs: 4.4–5.6%, *N* = 2), ChAd63/MVA (AEs: 0–100%, N = 6; SAE: 0.4–8.9%, *N* = 2), FMP2.1/AS02A (SAE: 4%, N = 1), GMZ2 (AEs: 23–100%, N = 2; SAEs: 49–54.5%, *N* = 2), PfPZ (AEs: 1.6–83.9%, *N* = 7; SAEs: 1.6%, *N* = 1), PfAMA1 (AEs: 5–60%, *N *= 1), PfSPZ-CVac (AEs: 19.4%, *N* = 1), Pfs25H-EPA (AEs: 100%, *N* = 1, SAEs: 1.7%, *N* = 1) and R21 (AEs 0.7–24.6%, *N* = 1, SAEs: 2.1%, *N* = 1).

The local and systemic AEs that were typically reported included injection site pain and fever among other symptoms including redness, warmth, discoloration, bruising, erythema, blistering, pruritis, swelling and induration; headache; allergic rash,; drowsiness; irritability; loss of appetite; fatigue; dizziness; abdominal pain; chills; myalgia; diarrhea; nausea and vomiting [18, 20, 30, 31, 37–39, 45, 46, 52, 56, 57, 59, 61–64, 66–70, 72, 74, 75, 77, 86–88]. Most AEs subsided within 1–7 days [18, 46, 52, 74, 86].

Commonly reported SAEs were acute gastritis, anemia, bronchitis, cerebral malaria, severe malaria, dehydration, convulsion, febrile convulsion, gastroenteritis, seizures, meningitis, paralytic ileus, pyrexia, pneumonia, respiratory distress, and death. However, most SAEs were.

deemed unrelated to the vaccination (Table 2) and were associated with malaria infection [29, 87]. Only 0.1–1% and 4.3% of SAEs were possibly linked to vaccines, mainly febrile convulsion/seizures, associated with RTS,S vaccine [25, 35, 58, 61–63, 66] and R21 vaccine [86] respectively. Malaria vaccine safety when co-administered with other routine childhood immunization was identified [20, 46].

**Table 2:** Safety of malaria vaccine

Malaria vaccine	Dose	References	Design	Safety issues			Comments
				Serious adverse events	Adverse events	None	
RTS,S (RTS,S/AS01, RTS,S/AS02) (V)	3 doses	Abdullah et al. [16]	RCT	✔			SAEs in 33.5% (95%CI 26.5–41.2) of infants
	3 doses	Asante et al. [20]	RCT	✔	✔		SAEs ranged from 5.1% to 6.1% of children. AEs in 1.6% (95%CI 0.8–2.9) to 82.7% (95%CI 77.2–87.3). Mainly pain and fever
	3 doses + 2 boosters	Chandramohan et al. [25]	RCT	✔			# SAEs incidence (events per 1000): cerebral malaria (0.2–0.7), severe malaria (2.0–6.7), severe malaria anemia (1.8–4.5), deaths (2.0–2.2). Vaccine-related SAE: 0.1% (5/3,474) cases of febrile seizures
	3–4 doses	Guerra Mendoza et al. [35]	RCT	✔			#SAEs in 24.2–28.4%. Vaccine-related SAEs 0.0–0.3%
	3 doses	Olotu et al. [55]	RCT	✔			SAEs in 17.9% (95% CI 13.1–23.6), notably severe malaria
	3–4 doses	Otieno et al. [58]	RCT	✔			#SAEs among 92.2% (95% CI 81.1–97.8) 4 dose; 85.2% (95%CI 72.9–93.4) 3 dose. SAEs include vaccine-related i.e., febrile convulsion. Groups involved HIV-infected children
	3 doses	RTS,S Clinical Trials partnership [61]	RCT	✔	✔		#SAEs in 17.9% (95% CI 16.8–19.1), 0.1% vaccine-related. AEs among 7.4–79.4%, mainly fever
	3 doses + booster	RTS,S Clinical Trials Partnership [62]	RCT	✔			#SAEs: 6–12 weeks group 22.0% (95%CI 20.8–23.3), 5–17 months group 18.6% (95%CI 17.6–19.6). 0.1–0.2% vaccine-related,
	3–4 doses	RTS,S Clinical Trials Partnership [63]	RCT	✔	✔		#SAEs in 24.2% (95%CI 22.7–25.8) to 26.6% (95%CI 24.8–28.5) 4 dose; 25.3% (95%CI 23.7–26.9) to 27.6% (95%CI 25.8–29.6) 3 dose; 0.1–0·3% vaccine-related. AEs among 1.5–36.3%, manly fever
	Variable doses 1–4 doses	Samuels et al. [66]	RCT	✔	✔		# SAEs from 15–20% across the standard regimen, full-dose, and fractional dose groups compared to control group (24%). Vaccine-related 1%. Major AEs (2–83%) include fever, pain
	3 doses	Umeh et al. [75]	RCT	✔	✔		SAEs in 2.8% (8/289). AEs were reported in a similar proportion of children in each group (72.5–87.5%) within 30 days post-vaccination, mainly pain and fever
	3 doses	Witte et al. [77]	RCT	✔	✔		SAEs in 6.7–16.7%. Unsolicited AEs in 50.9–82.5%; Solicited AEs incidence 0.6–17.2%, mainly pain, redness, swelling and fever
BK-SE36	2 doses	Palacpac et al. [59]	RCT	✔	✔		SAE (acute gastritis) 5.6%(1/18). AEs ranged from 5.6% (1/18) to 94.4% (17/8): mainly induration, pain, tenderness, fever
	3 doses	^a^Ouedraogo et al. [87]	RCT	✔	✔		SAE 4.4% (4/91). AEs 91% (83/91), mainly fever, pain, and diarrhea
ChAd63 ME-TRAP followed by MVA ME-TRAP	2 doses	Afolabi et al. [18]	RCT	✔	✔		SAE (gastroenteritis) in 1/24 participant (4.2%). AEs ranged from 3.3% to 50% mainly pain and fever
	2 doses	Mensah et al. [45]	RCT		✔		AEs in 1–35 (out of 57 vaccinees) post-vaccination; 1.8–61% (estimated from charts). Most common pain and fever
	2 doses	Mensah et al. [46]	RCT		✔		AEs ranged from 6.7–73.3%. Mainly fever, pain
	2 doses	Ogwang et al. [52]	RCT		✔		AEs ranged from 3.3% to 100%, mainly headache, pain
	2 doses	Tiono et al. [74]	RCT	✔	✔		SAEs (commonly reported): pneumonia (12 cases), non-severe malaria (8), gastroenteritis (4) and malnutrition (6) out of 336 vaccinees: 8.9%. AEs (estimated from chart) ranged from 1 to 48%, mainly pain, and fever
ChAd63 RH5 followed by MVA RH5	2 doses	^a^Silk et al. [88]	RCT		✔		AEs ranged from 0 to 100%, including swelling, pruritis, fever
FMP2.1/AS02A	3 doses	Laurens et al. [42]	RCT	✔			SAEs total: 4% (95%CI 1.9–7.9), 8/199 children
GMZ2 (GMZ2-CAF01, GMZ2-alum)	3 doses	Dassah et al. [29]	RCT	✔			SAEs ranged from 0.4% to 7.9% in 54.5% (91/167) vaccinees
	3 doses	Dejon-Agobe et al. [31]	RCT		✔		AEs: 496 recorded. Every volunteer had at least 1 AE/100% (range 1–29)
	3 doses	Sirima et al. [67]	RCT	✔	✔		SAEs in 49% (33/68) and 2 deaths. Local AEs 29% (268/925), general AEs 23% (216/925), mainly fever
PfSPZ	5 doses	Jongo et al. [37]	RCT		✔		AEs: 7.2% in 34.7% (17/49), including fever and headache
	3 doses	Jongo et al. [38]	RCT	✔	✔		SAE in 1.6% (1/63). AEs ranged from 1.6% (solicited) to 36.5% (unsolicited)
	3 doses	Olotu et al. [56]	RCT		✔		AEs in 5% (1/20), mainly fever, vomiting
	3 doses	Oneko et al. [57]	RCT		✔		AEs (solicited) in 51.6% (out of 252), mainly fever
	5 doses	Sissoko et al. [68]	RCT		✔		AEs in 2% (1/46) to 25% (3/12) of participants, mainly fever, headache and myalgia
	3 doses	Sissoko et al. [69]	RCT		✔		AEs ranged from 7 to 70%, (out of 60), mainly pain and headache
	2 variable doses	Steinhardt et al. [70]	RCT		✔		AEs ranged from 35.7% (solicited) to 83.9%(unsolicited) participants (out of 112), including fever and pain
PfAMA1-FVO [25–545]	3 doses	Thera et al. [72]	RCT		✔		AEs ranged from 5 to 60% of participants (out of 60), mainly injection site pain, headache and fever
PfSPZ-CVac	3 doses	Coulibaly et al. [27]	RCT		✔		AEs in 19.4%, (95%CI 9.2–36.3), 6/31participants
Pfs25H-EPA	4 doses	Sagara et al. [64]	RCT	✔	✔		SAEs in 1.7% (1/60); AEs (any) total: 929 in all participants, 60 (100%),137 (solicited) and 792 (unsolicited)
R21	3 doses + 1 booste	Datoo et al. [30]	RCT	✔	✔		SAE in 2.1% (6/292); solicited AEs range from 0.7% (1/138) to 24.6% (34/138) 7 days after booster
	3 doses + 1 booster	Datoo et al. [78]	RCT	✔			SAE in 1.3% (2/150) in low-dose and 2.0% (3/150) in high-dose groups 12 months after booster
	3 doses + 1 booster	^a^Datoo et al. [86]	RCT	✔	✔		#SAE in 2.7% (88/3252); Most common local AE, pain (18.6%), most common systemic AE, fever (46.7%)

Abbreviations and Notations—#: vaccine related SAE; *SAE* serious adverse event, *AE* adverse event, *BK-SE36* *Plasmodium* *falciparum* serine repeat antigen-5 formulated with aluminum hydroxyl gel, *ChAd63* *Chimpanzee Adenovirus 63* *CHMI* controlled human malaria infection, *CT* Clinical trial, *FMP2.1/AS02A* *Plasmodium* *falciparum* apical membrane antigen 1, *GMZ2: 2* *Plasmodium falciparum* antigens glutamate-rich protein and merozoite surface protein 3, *HLA-G* human leukocyte antigen G, *MSP* *Merozoite surface protein,* *MVA* modified vaccinia Ankara, *ME-TRAP* Multiple Epitope thrombospondin-related adhesion protein, *PfAMA1-FVO* Recombinant protein *Pichia pastoris*- expressed apical membrane antigen-1 from *Plasmodium falciparum* FVO clone adsorbed to Alhydrogel; Pfs25H-EPA: Recombinant pichia-expressed, His-tagged-Pfs25-conjugated to an *Escherichia coli*-expressed recombinant, *SMC* Seasonal malaria Chemoprevention, *EPA* *Pseudomonas aeruginosa* ExoProtein A, *PfSPZ* *Plasmodium* *falciparum* sporozoite, *PfSPZ-CVac* *Plasmodium* *falciparum* sporozoite chemoprophylaxis vaccine, *R21* Matrix-M/circumsporozoite protein-based Vaccine, *RCT* randomized controlled trial, *RR* risk ratio *RTS,S* recombinant protein malaria vaccine

^a^Article identified during peer-review

### Community perception of malaria vaccine

Seventeen studies (27.0%, *N* = 17) assessed community perception of malaria vaccines, with a mix of below and above-average quality studies (Table S2). The overall perception of participants has been summarized in addition to five key issues that emerged from the studies: acceptance, availability, knowledge/awareness, logistics, and misconceptions about the vaccines (Table 3).

**Table 3:** Community perception of malaria vaccine

Outcome	Data collection	References	Component			Key findings
			Vaccine nature	System	Personal	
Acceptance	FG and interviews	Achieng et al. [17]		✔	✔	Many children enrolled but were later removed due to factors such as objections to required blood draws
	Questionnaire	Asmare [21]			✔	32.3% (*n* = 406) respondents were willing to vaccinate their children
	FG and interviews	Bingham et al. [23]			✔	Participants said they would have their children vaccinated to keep them healthy
	Questionnaire	Chukwuocha et al. [26]			✔	95.6% (*n *= 500) positive intention to comply with vaccine
	Questionnaire	Etokidem et al. [33]		✔	✔	53.3% (*n* = 262) agreed that they would allow their children to be volunteers for malaria vaccine trial
	FG	Febir et al. [34]			✔	Participants agree to have their children vaccinated
	Questionnaire	Immurana et al. [36]			✔	94.6% (*n* = 3004) of the mothers are willing to allow their children to be given the malaria vaccine
	Questionnaire (scale)	Kpanake et al. [41]	✔	✔	✔	Acceptance positions include Neighbors’ Attitude (5%), Cost Only (21%), Neighbors’ Attitude and Cost (22%), Risk and Cost (33%), and Always Vaccine (20%)
	Questionnaire	McCoy et al. [43]	✔		✔	96% (*n* = 615) willing to accept the vaccine if it is safe and effective
	Questionnaire	Mtenga et al. [47]			✔	84.2% (*n* = 2123) mothers had perfect acceptance of malaria vaccine
	Questionnaire (online)	Musa et al. [48]			✔	67.9% (*n* = 131) would voluntarily allow their children to get vaccinated
	Questionnaire	Ojakaa et al. [53]		✔	✔	88% (*n* = 2003) indicated that they would accept a malaria vaccine
	Questionnaire	Romore et al. [60]			✔	94.5% (*n* = 5502) were willing to vaccinate their children
	Questionnaire (scale)	Vera Cruz et al. [76]	✔	✔	✔	Acceptance positions include cost (12%); Neighbors, risk, and cost (28%); Treatment, Risk, and cost (10%); always vaccinate (7%); risk and cost (13%); and Risk, Treatment, effectiveness, and cost (22%)
	Questionnaire	Sulaiman et al. [71]			✔	70.9% (*n* = 3389) not hesitant to accept vaccine
Availability	FG and interviews	Bingham et al. [23]		✔		Concern if vaccines will be available for adults in addition to children to ensure full protection
	Interview	McCoy et al. [43]		✔		Supply chain management problems led to loss of community interest based on prior vaccine shortages experience
Knowledge	FG and interviews	Achieng et al. [17]		✔	✔	Poor knowledge on malaria vaccine trials design
	Questionnaire	Asmare [21]			✔	Only 18% (*n* = 406) of caregivers were aware of the vaccine
	FG and interviews	Bingham et al. [23]			✔	The need to know how a future malaria vaccine would work, its duration of efficacy, dosage, potential side effects, who should receive the vaccine, and why
	Questionnaire	Chukwuocha et al. [26]			✔	48.2% (*n* = 500) aware of malaria vaccine
	Questionnaire	Etokidem et al. [33]			✔	60% (*n* = 262) heard about malaria vaccine prior to the study
	Questionnaire	Immurana et al. [36]			✔	40% (*n* = 3004) mothers aware of malaria vaccine
	Interview	Menaca et al. [44]			✔	Confusion between malaria vaccine and other childhood vaccines
	Questionnaire (online)	Musa et al. [48]			✔	56% (*n* = 236) of subjects ever heard about malaria vaccines
	Questionnaire	Romore et al. [60]			✔	11% (*n* = 5502) aware of malaria vaccine
Logistics	FG and interviews	Achieng et al. [17]		✔		Staff attitude and capacity as important considerations
	Questionnaire	Chukwuocha et al. [26]			✔	40.6% (*n* = 500) participants willing to pay for the vaccine
	Interview	McCoy et al. [43]		✔		Outreach by community health workers to encourage participation
	Interview	Darkwa et al. [28]	✔	✔	✔	Concerns about affordability remains, but participants were willing to pay (median USD 0.94), per dose of RTS,S/AS01, based on the belief that it is effective
Misconceptions	FG and interviews	Achieng et al. [17]		✔		Perception of blood theft and selling among parents/care givers
	Questionnaire and FG	McCoy et al. [43]	✔	✔	✔	Fears were a primary reason for unwillingness to receive vaccines e.g., infertility, government mistrust
	Questionnaire	Sulaiman et al. [71]	✔	✔		20.89% (*n* = 211) agreed they were hesitant because of a lack of trust in pharmaceutical companies, and 19.21% (*n* = 194) were afraid of the vaccine resulting in infertility
	FG and interviews	Bingham et al. [23]		✔	✔	Some study participants felt that community members may see a malaria vaccine as a sign that other prevention methods were no longer important
Overall perception	Questionnaire	Chukwuocha et al. [26]			✔	88.2% (*n* = 500), showed positive perception about the vaccine
	FG and interviews	Bingham et al. [23]			✔	Participants generally expected that a vaccine would help prevent malaria and allow children to lead healthy lives
	Questionnaire	Etokidem et al. [33]		✔	✔	84% (*n* = 262) believe malaria vaccine is necessary for malaria control. 86% (*n* = 262) recommend that malaria vaccine be made part of the country's National Programme on Immunization
	Questionnaire	Febir et al. [34]			✔	65.9% (*n* = 466) of respondents preferred vaccines to drugs for malaria control while 26.2% preferred drugs to vaccines
	Questionnaire	Immurana et al. [36]			✔	76.5% (*n* = 3004) mothers perceived most fever in children, as malaria (risk perception)
	Interview	Menaca et al. [44]			✔	Community members and health professionals agreed that it would be important to have a malaria vaccine. Mixed reaction on orals/injectables
	Interview and FG	Mtenga et al. [47]	✔		✔	Positive opinions towards malaria vaccine were due to a need for additional malaria prevention strategies and its expected benefits
	Questionnaire (online)	Musa et al. [48]			✔	72.5% (*n* = 131) knew that the vaccine could prevent malaria and 96.8% (*n* = 131) believe that the vaccine was necessary for the prevention of malaria
	Questionnaire	Romore et al. [60]		✔	✔	Most respondents would accept any proposed schedule (86.7%, *n* = 5502), or mode of administering the malaria vaccine (81.3%, *n* = 5502)
	Interview	Darkwa et al. [28]	✔	✔		Happy with services at vaccine trial coupled with perceived limited side effects. caregivers prefer vaccines over vector control measures

*FG* focus group; *n* number of participants

### Overall perception

Ten of the seventeen studies that assessed community perception (58.8%) reported their overall perception of malaria vaccines (Table 3), and were of below and above-average quality (Table S2). Community members agreed that it was essential to have a malaria vaccine [44] and that the vaccine is necessary for malaria control [33]. More than three-quarters of participants from each study reported overall positive perceptions [26, 36, 47, 48], identified malaria as a risk for their children [36], and identified that the vaccine will keep children healthy [23, 44] even though the efficacy of the vaccine may not be 100% [47]. A significant positive association between positive perception and intent to comply with vaccination was reported [26]. More than half of respondents recommend the vaccine to others [48] and were part of the National Program on Immunisation [33, 48]. The majority of participants preferred vaccines to malaria drugs/vector control [28, 34]. There was a mixed reaction between oral and injectable vaccines in Ghana [44], while in Tanzania, participants were open to all modes of administration [60]. The limited side effects experienced by participants in the RTS,S/AS01 vaccine trial reinforced participants’ beliefs about its safety in Nigeria [28].

### Acceptance

Of the studies examined, 88.2%, (*N* = 15) reported acceptance of malaria vaccines (Table 3), and most studies were above-average quality (Table S2). Acceptance rates varied from 32.3% in Ethiopia [21] to 96% in Sierra Leone [43]. Acceptance increased to 98.9% in malaria-endemic areas in Kenya [53]. Key drivers for acceptance were the high risk of malaria in children [17, 41], the desire for self-protection and prevention [41, 43], and incentives such as free consultations and medication [17].

The impact of religion on vaccine acceptance was inconsistent [36, 47, 71]. Some findings showed that Christian mothers were more likely to accept the vaccine than Muslim mothers in Tanzania [47], while in Ghana [36] and Nigeria [71], Christian mothers showed lower odds of accepting the vaccine. Free provision significantly increased vaccine acceptance [41, 43], while increased costs decreased acceptance [41, 76].

Fear of adverse events and unsuccessful intravenous vaccination attempts were linked to vaccine refusal [23, 43, 44, 71]. Factors such as marital status, region, knowledge of vaccine, tribe, education level, prior vaccination experience, satisfaction with healthcare services, and parent age influenced willingness to accept vaccination [21, 33, 41, 47, 53, 76].

### Availability

Two of the studies (11.8%) reported concerns associated with the availability of malaria vaccines (Table 3). The need to provide malaria vaccine to adults in addition to children was reported in Mozambique [23]. The importance of an adequate supply chain to promote availability was documented from a key informant interview in Sierra Leone [43].

### Knowledge/awareness

Nine of the studies (52.9%) reported knowledge of participants about malaria vaccines (Table 3). The percentage of participants having awareness of malaria vaccines ranged from 11% in Tanzania [60] to 60% in Nigeria [33]. Additionally, there was a low willingness to learn more about the vaccine in Mozambique [23]. Confusion and delays related to trial designs were seen to discourage participation in a malaria vaccine trial in Kenya [17]. The use of mass media, particularly Television, radio, and phones were identified as good sources of information by participants [23, 26, 44]. Information vans, health talks, and information from trusted community members [44] or health professionals were important but were rated equally with internet sources [71]. Awareness of vaccines was higher in older people when compared to younger people [36] and in mothers of Christian children compared to the Islamic faith [36]. There was evidence of confusion about malaria vaccines and other childhood vaccines in Ghana [44].

### Logistics

Four of the studies (23.5%) reported findings related to the logistics associated with malaria vaccine enrolments (Table 3). The need for community outreach by community health workers, including malaria vaccine campaigns alongside existing vector control programs to encourage participation was reported [43]. Negative attitudes of health staff were reported and shown to discourage participation in malaria vaccine trials [17]. Similarly, the system’s capacity to train staff for intravenous administration was noted as important [17].

Parents’ willingness to pay for the malaria vaccine was reported as a barrier [26, 28, 43]. Although, affordability was noted as a concern in a number of studies [26, 28, 41, 76], some participants suggested that the provision of malaria vaccines was the sole responsibility of the government [28].

### Misconceptions

Four of the studies (23.5%) reported misconceptions about potential malaria vaccines. Rumors of blood ‘theft and selling’ were linked to early withdrawal from malaria vaccine trials in Kenya [17]. Similarly, a widespread belief that newborns should have minimum exposure to adults and that the presence of a vaccine scar signifies a nurse had sexual intercourse with the child hindered vaccination programs in Mozambique [23]. The ideology that vaccines are harmful and can cause sickness was reported as a fear preventing vaccinations [23, 43]. Furthermore, rumors of vaccines causing infertility and system mistrust were cited as critical reasons for hesitancy to receive the malaria vaccine [43, 71].

## Discussion

This paper summarizes recent evidence on the efficacy, safety, and perception of malaria vaccines in Africa. All vaccines studied showed some degree of protection in terms of reducing the risk of contracting malaria and/or eliciting an antibody response. Overall efficacy varied; the highest overall efficacy (77%) was observed with R21 [30], which increased to 80% with a booster dose [78]. Increasing the dosage regimen of PfSPZ may also lead to an increase in efficacy from 20 to 100% [39]. Vaccination efficacy decreases over time with the highest efficacy expected up to one year after the last dose [55, 73]. R21 showed increased efficacy between six months (74%) to one year (77%) after vaccination [30]. RTS,S, was the most-studied vaccine. RTS,S showed good efficacy (55%) up to one year after vaccination, but this decreased over time [24, 55], with efficacy around zero after four years and negative in areas with high malaria exposure at five years of follow-up [55]. RTS,S was found to prevent clinical malaria cases in infants and children over three to four years and was further enhanced by administering a booster dose [63]. Emerging evidence suggests that the efficacy of vaccines like RTS,S increases when combined with seasonal malaria chemoprophylaxis [63]. The concomitant use of malaria vaccines with other control measures is therefore seen to be an important mitigation strategy in areas of high transmission.

Adverse events were reported in all studies. The most common adverse events were injection site pain and fever. Most adverse events were reported to subside within one week of appearance. Serious adverse events were rare (0.1–1%). Serious adverse events can occur following vaccinations, with about 1% of participants developing events such as febrile convulsions following malaria vaccines [23, 25, 35, 58, 61–63]. This was particularly observed in children within 2–3 days of receiving the RTS,S vaccine [35]. It is therefore possible that adverse events may arise following vaccination; however, further research is required.

Fear of unknown side effects associated with vaccines, especially newly developed ones, are often associated with low levels of acceptance [79]. Willingness to accept the malaria vaccine ranges from 32.3% in Ethiopia to 96% in Sierra Leone [21, 26]. However, a number of factors are likely to affect the use of malaria vaccines in many African communities, including inadequate knowledge, misconceptions, availability of vaccines, and logistics.

This review has identified that knowledge about malaria vaccines is not widespread throughout Africa. Vaccine awareness was slightly lower than vaccine acceptance; however, people may have been reluctant to accept the newly developed malaria vaccines because of generalized vaccine hesitancy in some parts of Africa. Vaccine hesitancy has been reported in the literature as a consequence of misinformation about vaccine origin, efficacy, and safety, and psychological factors such as anxiety [80, 81]. In addition to these factors, political influences, religious beliefs, and low perception of risk combine to contribute to vaccination rates in sub-Saharan Africa [79, 80]. The extent of vaccination hesitancy may vary according to people's commitment to health protection and risk culture and their trust in conventional medicine and public health authorities. Evidence from the literature suggests that the lack of willingness to vaccinate may be due to a lack of knowledge, indifference, and irregular vaccination behavior [82]. Public education campaigns on vaccination programs are therefore important to support behavior change.

The findings of this review could assist public health experts and policymakers in Africa to develop and implement strategies to address the low acceptance and use of malaria vaccines. Wide-spread adoption of malaria vaccines is possible if awareness campaigns provide adequate factual explanations to counter rumors and mis-information [6, 83]. Increasing local vaccine production within the African continent may further promote the use of malaria vaccines. Local production may help reduce mistrust through technology transfer. To raise awareness about vaccination, it is important to take a context-specific approach involving community and religious leaders [84, 85]. The provision of credible information to communities by trusted sources is an important strategy to promote vaccination uptake.

There are some limitations to this review. Due to recent advances in malaria vaccines and the recommendations of Schwartz et al. [9] only studies published since 2012 were included. The scope of this review summarizes the existing evidence and highlights areas for more in-depth analysis in the future.

## Conclusion

Different types of malaria vaccines have different efficacy levels, and combining seasonal malaria prophylaxis with a malaria vaccine might increase effectiveness. A variable degree of protection from malaria infection is provided by malaria vaccines with severe adverse events only occurring rarely. Many African communities have a high perception of malaria vaccines, but knowledge of the vaccine is relatively low. Further research and community involvement are needed to respectively improve vaccine efficacy and ensure successful widespread use in African communities.

## Supplementary Information

Below is the link to the electronic supplementary material.

Supplementary file1 (DOCX 100 KB)

## Data Availability

All data used in this review will be made available on request through the corresponding author.
