# Supplementary material for: Malaria vaccine efficacy, safety, and community perception in Africa: a scoping review of recent empirical studies
Source: Infection. 2024 Mar 5;52(5):2007–28. doi: 10.1007/s15010-024-02196-y (PMC11499420; doi:10.1007/s15010-024-02196-y)
Supplement: Supplementary file 1 — Supplementary file1 (DOCX 100 KB) [file 15010_2024_2196_MOESM1_ESM.docx]

**Journal:** INFECTION

**Malaria vaccine efficacy, safety, and community perception in Africa: a scoping review of recent empirical studies**

Muhammad Chutiyami^1*^. Priya Saravanakumar^1^. Umar Muhammad Bello^2^. Dauda Salihu^3^. Khadijat Kofoworola Adeleye^4^. Mustapha Adam Kolo^5^. Kabiru Kasamu Dawa^6^. Hamina Dathini^7^. Pratibha Bhandari^1^. Surajo Kamilu Sulaiman^8^. Jenny Sim^9,10^

1. School of Nursing and Midwifery, University of Technology Sydney, Sydney, Australia
2. Department of Physiotherapy and Paramedicine, School of Health and Life Sciences, Glasgow Caledonian University, Glasgow, United Kingdom
3. College of Nursing, Jouf University, Sakaka, Saudi Arabia
4. College of Nursing, University of Massachusetts, Amherst, MA 01003, USA
5. Department of Geography, University of Maiduguri, Maiduguri, Nigeria
6. School of Nursing, Midwifery and Health Education, University of Bedfordshire, Luton, United Kingdom
7. Department of Nursing Science, University of Maiduguri, Maiduguri, Nigeria
8. Department of Physiotherapy, Tishk International University, Iraq
9. WHO Collaborating Centre for Nursing, Midwifery & Health Development, University of Technology Sydney, Sydney, Australia
10. School of Nursing, Midwifery and Paramedicine, Australian Catholic University, Sydney, Australia

Correspondence: Muhammad Chutiyami, Muhammad.chutiyami@uts.edu.au

**Appendix 1: Preferred Reporting Items for Systematic reviews and Meta-Analyses extension for Scoping Reviews (PRISMA-ScR) Checklist**

| **SECTION** | **ITEM** | **PRISMA-ScR CHECKLIST ITEM** | **REPORTED ON PAGE #** |
| --- | --- | --- | --- |
| **TITLE** | | | |
| Title | 1 | Identify the report as a scoping review. | 1 |
| **ABSTRACT** | | | |
| Structured summary | 2 | Provide a structured summary that includes (as applicable): background, objectives, eligibility criteria, sources of evidence, charting methods, results, and conclusions that relate to the review questions and objectives. | 2 |
| **INTRODUCTION** | | | |
| Rationale | 3 | Describe the rationale for the review in the context of what is already known. Explain why the review questions/objectives lend themselves to a scoping review approach. | 3 |
| Objectives | 4 | Provide an explicit statement of the questions and objectives being addressed with reference to their key elements (e.g., population or participants, concepts, and context) or other relevant key elements used to conceptualize the review questions and/or objectives. | 3 |
| **METHODS** | | | |
| Protocol and registration | 5 | Indicate whether a review protocol exists; state if and where it can be accessed (e.g., a Web address); and if available, provide registration information, including the registration number. | 3 |
| Eligibility criteria | 6 | Specify characteristics of the sources of evidence used as eligibility criteria (e.g., years considered, language, and publication status), and provide a rationale. | 3 |
| Information sources* | 7 | Describe all information sources in the search (e.g., databases with dates of coverage and contact with authors to identify additional sources), as well as the date the most recent search was executed. | 4 |
| Search | 8 | Present the full electronic search strategy for at least 1 database, including any limits used, such that it could be repeated. | 4 |
| Selection of sources of evidence† | 9 | State the process for selecting sources of evidence (i.e., screening and eligibility) included in the scoping review. | 4 |
| Data charting process‡ | 10 | Describe the methods of charting data from the included sources of evidence (e.g., calibrated forms or forms that have been tested by the team before their use, and whether data charting was done independently or in duplicate) and any processes for obtaining and confirming data from investigators. | 4 |
| Data items | 11 | List and define all variables for which data were sought and any assumptions and simplifications made. | 5 |
| Critical appraisal of individual sources of evidence§ | 12 | If done, provide a rationale for conducting a critical appraisal of included sources of evidence; describe the methods used and how this information was used in any data synthesis (if appropriate). | 5 |
| Synthesis of results | 13 | Describe the methods of handling and summarizing the data that were charted. | 5 |
| **RESULTS** | | | |
| Selection of sources of evidence | 14 | Give numbers of sources of evidence screened, assessed for eligibility, and included in the review, with reasons for exclusions at each stage, ideally using a flow diagram. | 5 |
| Characteristics of sources of evidence | 15 | For each source of evidence, present characteristics for which data were charted and provide the citations. | 6 |
| Critical appraisal within sources of evidence | 16 | If done, present data on critical appraisal of included sources of evidence (see item 12). | 6 |
| Results of individual sources of evidence | 17 | For each included source of evidence, present the relevant data that were charted that relate to the review questions and objectives. | 6-9, Table 1, 2, 3 |
| Synthesis of results | 18 | Summarize and/or present the charting results as they relate to the review questions and objectives. | 6-9 |
| **DISCUSSION** | | | |
| Summary of evidence | 19 | Summarize the main results (including an overview of concepts, themes, and types of evidence available), link to the review questions and objectives, and consider the relevance to key groups. | 9 |
| Limitations | 20 | Discuss the limitations of the scoping review process. | 10 |
| Conclusions | 21 | Provide a general interpretation of the results with respect to the review questions and objectives, as well as potential implications and/or next steps. | 10 |
| **FUNDING** | | | |
| Funding | 22 | Describe sources of funding for the included sources of evidence, as well as sources of funding for the scoping review. Describe the role of the funders of the scoping review. | 11 |

JBI = Joanna Briggs Institute; PRISMA-ScR = Preferred Reporting Items for Systematic reviews and Meta-Analyses extension for Scoping Reviews.

**Supplemental Table S1: Literature search terms**

| Databases: Academic Search Complete, CINAHL, Medline with full-text, and PsychInfo | | | |
| --- | --- | --- | --- |
| Context  AND | Population  AND | Intervention  AND | Outcome |
| *Malaria* | Africa OR  “Sub-Saharan Africa” OR SSA OR  “Low and middle-income countries” OR LMIC OR  Endemic OR  Algeria OR  Angola OR  Benin OR  Botswana OR  “Burkina Faso” OR  Burundi OR  Cameroon OR  “Cape Verde” OR  “Central Africa Republic” OR  Chad OR  Comoros OR  “Democratic Republic of Congo” OR  “Republic of Congo” OR  “Cote d’Ivoire” OR  Djibouti OR  Egypt OR  “Equatorial Guinea” OR  Eritrea  Ethiopia OR  Gabon OR  Gambia OR  Ghana OR  Guinea OR  “Guinea-Bissau” OR  Kenya OR  Lesotho OR  Liberia OR  Libya OR  Madagascar OR  Malawi OR  Mali OR  Mauritius OR  Morocco OR  Mozambique OR  Namibia OR  Niger OR  Nigeria OR  Rwanda OR  “Sao Tome and Principe” OR Senegal OR  Seychelles OR  “Sierra Leone” OR  Somalia OR  “South Africa” OR Sudan OR  “South Sudan” OR  Swaziland OR  Tanzania OR  Togo OR  Tunisia OR Uganda OR Zambia OR Zimbabwe | Vaccine* OR  “Malaria vaccine*” OR  Immun* | Efficacy OR effect* OR  Safety OR react* OR  Perception OR view OR  accept* |
| Database: African Journals Online (AJOL) | | | |
| 1^st^ search | General index terms | malaria vaccine efficacy, safety, perception | |
| 2^nd^ search | Specific index terms | Efficacy: Malaria vaccine efficacy  Safety: Malaria vaccine safety  Perception: Malaria vaccine perception | |

**Supplemental Table S2: Quality appraisal of included studies**

| Table S2a: Quality of Randomised Controlled Trials (RCT) using JBI tool | | | | | | | | | | | | | | | |
| --- | --- | --- | --- | --- | --- | --- | --- | --- | --- | --- | --- | --- | --- | --- | --- |
| Study | 1 | 2 | 3 | 4 | 5 | 6 | 7 | 8 | 9 | 10 | 11 | 12 | 13 | Total | Quality interpretation |
| Abdullah et al. [17] | Y | Y | Y | Y | U | N | Y | Y | Y | Y | Y | Y | Y | 11/13 | Above-average |
| Afolabi et al. [19] | Y | Y | Y | Y | N | Y | Y | Y | Y | Y | Y | Y | Y | 12/13 | Above-average |
| Ajua et al. [20] | Y | Y | Y | N | N | N | Y | Y | Y | Y | Y | Y | Y | 10/13 | Above-average |
| Asante et al. [21] | Y | Y | Y | U | U | U | Y | Y | Y | Y | Y | Y | Y | 10/13 | Above-average |
| Bejon et al. [23] | Y | Y | Y | U | U | U | Y | Y | Y | Y | Y | Y | Y | 10/13 | Above-average |
| Cairns et al. [25] | Y | Y | Y | Y | Y | Y | Y | Y | Y | Y | Y | Y | Y | 13/13 | Above-average |
| Chandramohan et al. [26] | Y | Y | Y | U | U | U | Y | Y | Y | Y | Y | Y | Y | 10/13 | Above-average |
| Coulibaly et al. [28] | Y | Y | Y | Y | Y | Y | U | Y | Y | Y | Y | Y | Y | 12/13 | Above-average |
| Dassah et al. [30] | Y | Y | Y | Y | Y | Y | Y | Y | Y | Y | Y | Y | Y | 13/13 | Above-average |
| Datoo et al. [31] | Y | Y | Y | Y | Y | Y | Y | Y | Y | Y | Y | Y | Y | 13/13 | Above-average |
| Datoo et al. [79] | Y | Y | Y | Y | Y | Y | Y | Y | Y | Y | Y | Y | Y | 13/13 | Above-average |
| Datoo et al. [87] | Y | Y | Y | Y | Y | Y | Y | Y | Y | Y | Y | Y | Y | 13/13 | Above-average |
| Dejon-Agobe et al. [32] | U | U | Y | N | U | U | Y | Y | Y | Y | Y | Y | Y | 08/13 | Above-average |
| Guerra Mendoza et al. [36] | Y | U | Y | U | Y | Y | Y | Y | Y | Y | Y | Y | Y | 11/13 | Above-average |
| Jongo et al. [38] | Y | Y | Y | Y | Y | Y | Y | Y | Y | Y | Y | Y | Y | 13/13 | Above-average |
| Jongo et al. [39] | Y | Y | Y | Y | N | U | Y | Y | Y | Y | Y | Y | Y | 11/13 | Above-average |
| Jongo et al.[40] | Y | Y | Y | Y | Y | Y | Y | Y | Y | Y | Y | Y | Y | 13/13 | Above-average |
| Jongo et al. [41] | Y | U | Y | Y | Y | U | Y | Y | Y | Y | Y | Y | Y | 11/13 | Above-average |
| Laurens et al. [43] | Y | Y | Y | Y | N | Y | Y | Y | Y | Y | Y | Y | Y | 12/13 | Above-average |
| Mensah et al. [46] | Y | Y | Y | Y | Y | Y | Y | Y | Y | Y | Y | Y | Y | 13/13 | Above-average |
| Mensah et al. [47] | Y | Y | Y | N | N | Y | Y | Y | Y | Y | Y | Y | Y | 11/13 | Above-average |
| Naefsey et al. [50] | Y | Y | Y | U | U | U | Y | Y | Y | Y | Y | Y | Y | 10/13 | Above-average |
| Nouatin et al. [51] | Y | U | Y | Y | Y | Y | Y | Y | Y | Y | Y | Y | Y | 12/13 | Above-average |
| Nouatin et al. [52] | Y | Y | Y | Y | Y | Y | Y | Y | Y | Y | Y | Y | Y | 13/13 | Above-average |
| Ogwang et al. [53] | Y | Y | U | N | N | N | N | Y | Y | Y | Y | Y | Y | 8/13 | Above-average |
| Olotu et al. [55] | Y | Y | Y | NA | Y | Y | Y | Y | Y | Y | Y | Y | Y | 12/13 | Above-average |
| Olotu et al. [56] | Y | Y | Y | U | U | U | Y | Y | Y | Y | Y | Y | Y | 10/13 | Above-average |
| Olotu et al. [57] | Y | Y | Y | Y | Y | Y | Y | Y | Y | Y | Y | Y | Y | 13/13 | Above-average |
| Oneko et al. [58] | Y | Y | Y | Y | Y | Y | Y | Y | Y | Y | Y | Y | Y | 13/13 | Above-average |
| Otieno et al. [59] | Y | Y | Y | U | U | Y | Y | Y | Y | Y | Y | Y | Y | 11/13 | Above-average |
| Ouedraogo et al [88] | Y | Y | Y | Y | Y | Y | Y | Y | Y | Y | Y | Y | Y | 13/13 | Above-average |
| Palacpac et al. [60] | Y | Y | Y | Y | Y | Y | Y | Y | Y | Y | Y | Y | Y | 13/13 | Above-average |
| RTSS Clinical Trial Partnership [62] | Y | Y | Y | U | U | U | Y | Y | Y | Y | Y | Y | Y | 10/13 | Above-average |
| RTSS Clinical Trial Partnership [64] | Y | U | Y | U | U | U | Y | Y | Y | Y | Y | Y | Y | 9/13 | Above-average |
| RTSS Clinical Trial Partnership [63] | Y | U | Y | U | U | U | Y | Y | Y | Y | Y | Y | Y | 9/13 | Above-average |
| Sagara et al. [65] | Y | Y | Y | Y | Y | Y | Y | Y | Y | Y | Y | Y | Y | 13/13 | Above-average |
| Sagara et al. [66] | Y | U | Y | U | U | U | N | Y | Y | Y | Y | Y | Y | 8/13 | Above-average |
| Samuels et al. [67] | Y | Y | Y | NA | N | N | Y | Y | Y | Y | Y | Y | Y | 10/13 | Above-average |
| Silk et al. [89] | Y | Y | Y | Y | Y | Y | Y | Y | Y | Y | Y | Y | Y | 13/13 | Above-average |
| Sirima et al. [68] | Y | Y | Y | Y | U | U | Y | Y | Y | Y | Y | Y | Y | 11/13 | Above-average |
| Sissoko et al. [70] | Y | Y | Y | Y | Y | Y | N | Y | Y | Y | Y | Y | Y | 12/13 | Above-average |
| Sissoko et al. [69] | Y | Y | Y | U | U | U | Y | Y | Y | Y | Y | Y | Y | 10/13 | Above-average |
| Steinhardt et al. [71] | Y | Y | Y | Y | N | Y | Y | Y | Y | Y | Y | Y | Y | 12/13 | Above-average |
| Thera et al. [73] | Y | Y | Y | Y | Y | U | Y | Y | Y | Y | Y | Y | Y | 12/13 | Above-average |
| Tinto et al. [74] | Y | N | Y | U | U | U | Y | Y | Y | Y | Y | Y | Y | 9/13 | Above-average |
| Tiono et al. [75] | Y | Y | Y | Y | U | U | Y | Y | Y | Y | Y | Y | Y | 11/13 | Above-average |
| Umeh et al. [76] | Y | Y | Y | Y | U | U | Y | Y | Y | Y | Y | Y | Y | 11/13 | Above-average |
| Witte et al. [78] | Y | U | Y | U | U | N | N | Y | Y | Y | Y | Y | Y | 8/13 | Above-average |
| JBI tool items:  1. Was true randomization used for the assignment of participants to treatment groups?  2. Was allocation to treatment groups concealed?  3. Were treatment groups similar at the baseline?  4. Were participants blind to treatment assignment?  5. Were those delivering treatment blind to treatment assignment?  6. Were outcomes assessors blind to treatment assignment?  7. Were treatment groups treated identically other than the intervention of interest?  8. Was follow-up complete and if not, were differences between groups in terms of their follow up adequately described and analyzed?  9. Were participants analyzed in the groups to which they were randomized?  10. Were outcomes measured in the same way for treatment groups?  11. Were outcomes measured in a reliable way?  12. Was appropriate statistical analysis used?  13. Was the trial design appropriate, and any deviations from the standard RCT design (individual randomization, parallel groups) accounted for in the conduct and analysis of the trial? | | | | | | | | | | | | | | | |

*Key:* Y, yes; N, no; U, unclear; NA, not applicable.

| Table S2: Quality of Case-Control Study using JBI tool | | | | | | | | | | | | |
| --- | --- | --- | --- | --- | --- | --- | --- | --- | --- | --- | --- | --- |
| Study | 1 | 2 | 3 | 4 | 5 | 6 | 7 | 8 | 9 | 10 | Total | Quality interpretation |
| Dobano et a. [33] | Y | Y | Y | Y | Y | Y | Y | Y | Y | Y | 10/10 | Above-average |
| JBI tool items;  1. Were the groups comparable other than the presence of disease in cases or the absence of disease in controls?  2. Were cases and controls matched appropriately?  3. Were the same criteria used for identification of cases and controls?  4. Was exposure measured in a standard, valid and reliable way?  5. Was exposure measured in the same way for cases and controls?  6. Were confounding factors identified?  7. Were strategies to deal with confounding factors stated?  8. Were outcomes assessed in a standard, valid and reliable way for cases and controls?  9. Was the exposure period of interest long enough to be meaningful?  10. Was appropriate statistical analysis used? | | | | | | | | | | | | |

Key: Y=yes, N=no, U=unclear/can’t tell

| Table S2c: Quality of cross-sectional studies/surveys using JBI tool | | | | | | | | | | |
| --- | --- | --- | --- | --- | --- | --- | --- | --- | --- | --- |
| Study | 1 | 2 | 3 | 4 | 5 | 6 | 7 | 8 | Total | Quality interpretation |
| Asmare [22] | Y | Y | Y | Y | Y | Y | Y | Y | 8/8 | Above-average |
| Chukwuocha et al. [27] | Y | Y | Y | Y | U | U | Y | Y | 6/8 | Above-average |
| Etokidem et al. [34] | Y | Y | Y | U | N | N | U | Y | 4/8 | Below-average |
| Imuranna et al. [37] | N | Y | Y | Y | N | N | Y | Y | 5/8 | Above-average |
| Kpnake et al. [42] | Y | Y | Y | Y | U | N | Y | Y | 6/8 | Above-average |
| Musa et al. [49] | Y | N | U | U | N | N | Y | Y | 3/8 | Below-average |
| Ojakaa et al. [54] | Y | Y | Y | Y | N | N | Y | Y | 6/8 | Above-average |
| Romore et al. [61] | Y | Y | Y | Y | U | U | Y | Y | 6/8 | Above-average |
| Sulaiman et al. [72] | Y | Y | Y | Y | U | N | Y | Y | 6/8 | Above-average |
| Vera Cruz et al. [77] | Y | Y | Y | Y | U | U | Y | Y | 6/8 | Above-average |
| JBI tool items:  1. Were the criteria for inclusion in the sample clearly defined?  2. Were the study subjects and the setting described in detail?  3. Was the exposure measured in a valid and reliable way?  4. Were objective, standard criteria used for measurement of the condition?  5. Were confounding factors identified?  6. Were strategies to deal with confounding factors stated?  7. Were the outcomes measured in a valid and reliable way?  8. Was appropriate statistical analysis used? | | | | | | | | | | |

*Key:* Y, yes; N, no; U, unclear; NA, not applicable.

| Table 2d: Quality of qualitative studies using JBI tool | | | | | | | | | | | | |
| --- | --- | --- | --- | --- | --- | --- | --- | --- | --- | --- | --- | --- |
| Study | 1 | 2 | 3 | 4 | 5 | 6 | 7 | 8 | 9 | 10 | Total | Quality interpretation |
| Achieng et al. [18] | Y | Y | Y | Y | Y | Y | U | Y | Y | Y | 9/10 | Above-average |
| Bingham et al. [24] | Y | Y | Y | Y | Y | Y | U | Y | Y | Y | 9/10 | Above-average |
| Darkwa et al. [29] | Y | Y | Y | Y | Y | Y | Y | Y | Y | Y | 10/10 | Above-average |
| Menaca et al. [45] | Y | Y | Y | Y | Y | Y | Y | Y | Y | Y | 10/10 | Above-average |
| JBI tool items:  1. Is there congruity between the stated philosophical perspective and the research methodology?  2. Is there congruity between the research methodology and the research question or objectives?  3. Is there congruity between the research methodology and the methods used to collect data?  4. Is there congruity between the research methodology and the representation and analysis of data?  5. Is there congruity between the research methodology and the interpretation of results?  6. Is there a statement locating the researcher culturally or theoretically?  7. Is the influence of the researcher on the research, and vice- versa, addressed?  8. Are participants, and their voices, adequately represented?  9. Is the research ethical according to current criteria or, for recent studies, and is there evidence of ethical approval by an appropriate body?  10. Do the conclusions drawn in the research report flow from the analysis, or interpretation, of the data? | | | | | | | | | | | | |

*Key:* Y, yes; N, no; U, unclear; NA, not applicable.

| Table Se: Quality of Mixed-Method Studies using MMAT | | | | | | | |
| --- | --- | --- | --- | --- | --- | --- | --- |
| Studies | 1 | 2 | 3 | 4 | 5 | Total | Quality interpretation |
| Febir et al. [35] | Y | Y | Y | U | Y | 4/5 | Above-average |
| McCoy et al.[44] | Y | Y | Y | Y | Y | 5/5 | Above-average |
| Mtenga et al. [48] | Y | Y | Y | Y | Y | 5/5 | Above-average |
| MMAT tool items:   1. Is there an adequate rationale for using a mixed methods design to address the research question? 2. Are the different components of the study effectively integrated to answer the research question? 3. Are the outputs of the integration of qualitative and quantitative components adequately interpreted? 4. Are divergences and inconsistencies between quantitative and qualitative results adequately addressed? 5. Do the different components of the study adhere to the quality criteria of each tradition of the methods involved? | | | | | | | |

Key: Y=yes, N=no, U=unclear/can’t tell

**Supplemental Table S3: Characteristics of included studies**

| Author(s) | Study country | Population | Sample size | Participants’ age/gender | Study design | Sampling | Outcome(s) assessed | Outcome measure(s) | Malaria vaccine(s) |
| --- | --- | --- | --- | --- | --- | --- | --- | --- | --- |
| Abdulla et al. [17] | Tanzania | Infants | 340 | Age (0-20 months)  Gender- NR | RCT | NR | 1. Safety outcomes  2. Immunogenicity outcomes  3. Efficacy outcomes | 1. Severe adverse effect  2. Anti-CS response  3. Incidence of first or only malaria episodes | RTS,S/  AS02_D_ |
| Achieng et al. [18] | Kenya | 1. Primary caregivers (i.e., mothers and fathers) of eligible infants  2. Community members (i.e., village elders)  3. Study clinicians | IDI- 6  FGD (mothers in trial-49, Mothers who withdrew 36) | 1. Primary caregivers  Mothers in trial [30.3 (20–42)]  Mothers who withdrew [30.8 (20–47)]  Fathers who withdrew [32.8 (27-46)]  2. Community members (i.e., village elders) [39.2 (34–45)]  3. Study clinicians [35.3 (33–38)] | Qualitative study | Purposive sampling | 1. Perceptions of the DVI procedure in infants    2. Factors influencing trial acceptability  3.Barriers to sustained trial participation | NR | PfSPZ |
| Afolabi et al. [19] | Gambia and Burkina Faso | Children  Infants | 138 | The Gambia  (10 weeks; 5–12 months & 2–6 years)  Burkinafaso  (5–17 months) | RCT | NR | 1. Safety  2.  Immunogenicity | 1. Adverse event  2. Increase in METRAP-specific IFNγ-secreting T-cells as measured by EnzymeLinked ImmunoSpot (ELISPOT) assay | ChAd63 and MVA ME-TRAP |
| Ajua et al. [20] | Gabon, Ghana and Tanzania | Infants | 511 | 0-7 months | RCT | Random sampling | 1. Protective efficacy and  2.  Immunogenicity | 1. Antibody measurement using ELISA    2. Anti-circumsporozoite protein antibody avidity | RTS,S/AS01E |
| Asante et al. [21] | Ghana | Children | N= 709 | Healthy children aged 6 months at the time of first vaccination | RCT | Centralized randomization system. | 1.  Immunogenicity  2. Safety | 1. Anti-CS antibody seropositivity rates  2. Serious adverse events | RTS,S/AS01 |
| Asmare [22] | Ethiopia | Caregivers of under-5 children | N= 406 | Male and female less than 60 years | Cross-sectional study | Proportionate sampling | 1. Willingness to accept malaria vaccine  2. Knowledge about malaria vaccine | NR | NR |
| *Bejon et al. [23] | Gambia  Mozambique  Tanzania  Gabon  Ghana  Kenya | Healthy adults or children | N= 4453 | Adults (NR)  Children (younger than 6 years) | RCT | NR | 1. Vaccine efficacy | Clinical malaria episodes | RTS,S |
| Bingham et al. [24] | Mozambique | 1 Caregivers  2. Community leaders  3. Heads of local service units and NGOs leaders. | N= 200  FGD- 23 and  IDI - 26 | NR | Qualitative study | Criterion-based sampling | 1. Perceptions, beliefs, attitudes, and health practices held in relation to malaria and vaccines | NR | NR |
| *Cairns et al. [25] | Mali  Burkina Faso | Children | N= 5920 | Children aged 5–17 months | RCT | NR | Protective efficacy | Protection against clinical malaria | RTS,S/AS01E |
| Chandramohan et al. [26] | Burkina Faso  Mali | Children | N= 6861 | Children 5 to 17 months of age | RCT | Randomization list used permuted blocks | 1. Vaccine efficacy  2. Vaccine safety | 1. Clinical malaria among the children  2. Adverse event | RTS,S/AS01E |
| Chukwuocha et al.[27] | Nigeria | Care givers  (mothers) | 500 | Female  Mean age (19.53±7.24 years) | Cross sectional descriptive design | Cluster sampling | 1. Awareness about malaria and the prospective malaria vaccine  2. Perception about malaria vaccine  3. Intent to comply with the prospective malaria vaccine | NR | NR |
| Coulibaly et al. [28] | Mali | Healthy, malaria-exposed adult men and non-pregnant women | N= 62 | aged 18-45 years old  1. Experiment mean age 31.5 (8-9)  2. Control group, mean age 29.3 (8-10) | RCT | computer-based enrolment module | 1. Safety  2. Vaccine efficacy | 1. adverse events  2. Plasmodium falciparum detection | PfSPZ-CVac |
| Darkwa et al. [29] | Ghana | Caregivers of children | N= 20 | caregivers of children aged 6–24 months | Qualitative study | Purposive sampling | caregivers’ willingness to  pay for the malaria vaccine | NR | RTS,S/AS01 |
| Dassah et al. [30] | Gabon  Burkina Faso  Ghana  Uganda | Children | N= 1849  Gabon (n = 512), Burkina Faso (Banfora; n = 580, Sapone; n = 300), Ghana (n = 200) and Uganda (n = 257) | children aged 12–60 months | RCT | block randomisation | 1. Vaccine efficacy against P. falciparum  2. Safety | 1. clinical malaria episodes  2. Adverse event | GMZ2/alum |
| Datoo et al. [31] | Burkina Faso | Children | N= 450 | children aged 5–17 months | RCT | block randomisation | 1. Vaccine efficacy  2. Safety, | 1. clinical malaria  2. Adverse events | R21/MM |
| Datoo et al. [79] | Burkina Faso | Children | N= 408 | children aged 5–17 months | RCT | block randomisation | 1. Vaccine efficacy  2. Safety, | 1. clinical malaria  2. Serious adverse event | R21/MM |
| Datoo et al. [87] | Burkina Faso, Mali, Kenya, Tanzania | Children | 4800 | Children aged 5- 36 months | double-blind, randomised, controlled trial | Stratified random sampling | 1. Safety outcomes  2. Efficacy outcomes  3. Immunogenicity | 1. Serious Adverse Events  2. Clinical malaria  3. IgG antibodies | RTS,S/AS01 |
| Dejon-Agobe et al. [32] | Gabon | Adults | N= 50 | Adults | RCT | NR | 1. Vaccine safety  2. Vaccine efficacy  3. Immunogenicity | 1. Adverse event  2. parasitemia level and symptoms  3. GMZ2-, GLURP-, and MSP3-specific IgG | GMZ2  MSP3 |
| Dobaño et al. [33] | Ghana  Mozambique | Infants  Children | N=195 | infants (6–12 weeks) and children (5–17 months) | Case control study | Randomly | antibody responses to vaccine unrelated Plasmodium falciparum | IgM and IgG responses to 38 P. falciparum proteins | RTS,S/AS01E |
| Etokidem et al. [34] | Nigeria | Adults | N= 262 | NR | descriptive cross-sectional study | systematic random sampling | 1. Perception of Malaria vaccine  2. Acceptability of Malaria vaccine | NA | NR |
| Febir et al. [35] | Ghana | Adults | Cross sectionalN= 466  FGD= 12  IDI= 15 | Adult with age range 29-39 | Mixed method study | Quantitative method: systematic sampling  Qualitative method: Purposive sampling | 1. Knowledge of vaccines  2. Perception of Malaria vaccine  3. | NR | NR |
| Guerra Mendoza et al. [36] | Burkina Faso, Ghana, Gabon, Kenya, Tanzania, Malawi, and Mozambique | Infants  Children | N= 15, 459 | Children (5–17 months)  infants (6–12 weeks) | RCT | Block randomisati+on | 1. safety | 1. Adverse event | RTS,S/AS01 |
| Immurana et al. [37] | Ghana | Women | N= 3004 | All women between ages 15–49 | Retrospective observational study | Proportionate sampling | Determinants of willingness to accept child vaccination against malaria | NR | NR |
| Jongo et al. [38] | Tanzania | Adults | N= 67 | male (18–35 years) | RCT | NR | 1. Safety,  2.Immunogenicity  3. Protective Efficacy | 1. Adverse event  2. Antibody assays and T-cell assays  Controlled human malaria infection | PfSPZ Vaccine |
| Jongo et al. [39] | Tanzania | Adults, Adolescents, Children, and Infants | N= 173 | male and female volunteers (6 months to 45 years) | RCT | Sequential allocation | vaccine safety  Differential Antibody responses | Adverse event  T-cell responses | PfSPZ Vaccine |
| Jongo et al. [40] | Tanzania | Adults | N= 30 | healthy males and females aged 18–45  years | RCT | Sequential allocation | Vaccine efficiency. | parasitemia by quantitative polymerase chain reaction | PfSPZ Vaccine |
| Jongo et al. [41] | Tanzania | Adults | N= 39 | Adults (18-35 years) | RCT | NR | Immunogenicity  Protective Efficacy | Antibody responses  first positive quantitative PCR result | PfSPZ Vaccine |
| Kpanake et al. [42] | Togo | Parents of Infants | N= 209 | Infants (0-12 months) | Cross sectional | Convenient sampling | Potential acceptability | NR | NR |
| Laurens et al. [43] | Mali | Children | N= 400 | Children 1-6 years | RCT | computer-generated predefined block randomization | 1. safety  2.immunogenicity and  3. efficacy | 1. Serious adverse event  2. anti-AMA1 immune responses  3. Clinical episode of malaria | FMP2.1/AS02A |
| McCoy et al. [44] | Sierra Leone | Adults | Cross sectional N= 615  FGD N= 6  IDI N=20 | Adult greater than 18 years | Mixed method study | Random location generator | 1. knowledge and health behaviours  2. Vaccine attitudes and acceptability | NR | NR |
| Menaca et al. [45] | Ghana | Adults | FGD N=25  IDI N= 107  semi-structured observations N = 21 | NR | Qualitative study | Purposive sampling | Factors affecting community acceptance of Malaria Vaccine | NR | NR |
| Mensah et al. [46] | Senegal | Adults | N= 120 | Adult (Mean age, 30.2) | RCT | Computer generated randomisation | 1. Safety  2.Immunogenicity  3. Efficacy | 1. Adverse event  2. IgG ELISA and ex-vivo interferon-gamma (IFNγ) ELISpot  3. Detection of malaria parasites in blood by quantitative PCR (qPCR) assays. | ChAd63 and  MVA Encoding ME-TRAP |
| Mensah et al. [47] | Gabon | Infants  Neonates | N= 65 | NR | RCT | Block randomization | 1. Safety  2.Immunogenicity | 1. Adverse event  2. IFNγ enzyme-linked immunospot, wholeblood flow cytometry, and anti-TRAP IgG ELISA | ChAd63  And  ME-TRAP |
| Mtenga et al. [48] | Tanzania | Mothers of children under five years | Cross sectional N= 2123  FGD N= 12  IDI N= 46 | Mean age = 28.9 ± 7.6 | Mixed methods | Purposive sampling | 1. acceptance of the anticipated malaria vaccine  2. perception and attitude towards the vaccines | NR | NR |
| Musa et al. [49] | Nigeria | Women | N= 236 | Women aged 15-49 | Cross sectional study | total population sampling | Awareness, Perception and Acceptance | NR | NR |
| Neafsey et al. [50] | Burkina Faso  Ghana  Gabon  Mozambique  Malawi  Tanzania  Kenya | Children | N= 6912 | children 5 to 17 months of age | RCT | NR | 1. Genetic Diversity  2. Protective Efficacy | 1. The circumsporozoite protein C-terminal and SERA-2 amplicons were sequenced on an Illumina MiSeq platform  2. clinical episodes of malaria infection | RTS,S/AS01 |
| Nouatin et al. [51] | Gabon | Adults | N= 50 | Adult 18-40 years | RCT | NR | 1. Vaccine efficacy    2. Immuninogenesity | Vaccine efficacy was assessed using controlled human malaria infection  Detection of anti-GMZ2 total IgG | GMZ2 |
| Nouatin et al. [52] | Gabon | Adults | N= 50 | NR | RCT | NR | 1. Vaccine efficacy  2. Cellular and antibody response  3. Safety | 1. Vaccine efficacy was assessed using controlled human malaria infection  2. Circulating B cell response to GMZ2  GMZ2 stimulated CD4+ T cells response  Adverse event | GMZ2 |
| Ogwang et al. [53] | Gambia  Kenya | Adults | N= 46 | Healthy males aged 18–50 years | RCT | systematic allocation | 1. Safety and  2. Immunogenicity | Adverse event  T cell responses | ChAd63 ME-TRAP  MVA METRAP |
| Ojakaa et al. [54] | Kenya | Adults | N= 2,003 | Adult male and female 20-34 years | Survey | Stratified sampling | Acceptance of a malaria vaccine | NR | NR |
| Olotu et al. [55] | Kenya  Tanzania | Children | N= 447 | Healthy children who were 5 to 17 months of age | RCT | NR | Four years efficacy | Infection with Plasmodium falciparum of >2500 parasites per cubic millimeter | RTS,S/AS01E |
| Olotu et al.[56] | Kenya  Tanzania | Children | N= 447 | Healthy children who were 5 to 17 months of age | RCT | NR | Seven years efficacy | Infection with Plasmodium falciparum of >2500 parasites per cubic millimeter | RTS,S/AS01 |
| Olotu et al. [57] | Equatorial Guinea | Adults | N= 33 | Healthy male adults between 18 and 35 years of age | RCT | Computer generated random numbers | 1.Safety  2.Immunogenicity | 1. Adverse event  3. IgG antibodies response | PfSPZ |
| Oneko et al. [58] | Kenya | Infants | N= 336 | infants aged 5–12 months | RCT | NR | 1. Safety  2.immunogenicity  3. efficacy | 1. Adverse event  2. T cell responses in infant  3. parasitemia by blood smear and a single quantitative PCR | PfSPZ |
| Otieno et al. [59] | Burkina Faso, Gabon, Ghana, Kenya, Malawi, Mozambique and Tanzania | Infants Children | N= 15,459 | Infants and children between 6 weeks to 17 months | RCT | NR | 1. Safety  2.immunogenicity | 1. Severe adverse events  2. seropositive for anti-CS antibodies in both age categories | RTS,S/AS01 |
| Ouedraogo et al. [88] | Burkina Faso | Adults  Children | 135 | children aged 7 to 10 years | double-blind, randomised, controlled | Sequentially numbered opaque, sealed envelopes | 1. Safety  2. immunogenicity | Adverse Events Following Immunisation (AEFI)  IgG titre | BK-SE36/CpG |
| Palacpac et al. [60] | Uganda | Adults | N= 56 | healthy adults aged 21–40 years | RCT | computer-generated sequence | 1. Safety  2.immunogenicity | 1. Serious adverse events  2. Anti-SE36 IgG | BK-SE36 |
| Romore et al. [61] | Tanzania | Women  Children | N= 5502 | women, aged 18 years or older and with children under 11 months old | cross-sectional study | Cluster sampling | 1 Awareness to use malaria vaccine  2. Willingness to use malaria vaccine | NR | NR |
| RTSS Clinical Trial Partnership. [62] | NR | Infants | N= 6537 | Infants 6-12 weeks | RCT | NR | 1. safety  2.immunogenicity  3. Efficacy | 1. Serious adverse events  2. Anti-circumsporozoite antibodies  Diagnosis of severe malaria | RTS,S/AS01 |
| RTSS Clinical Trial Partnership. [63] | Burkina Faso  Ghana  Gabon  Kenya  Malawi  Tanzania  Mozambique | Infants  children | N= 6537 | infants aged 6–12 week and children aged 5–17 months | RCT | block randomisation | 1. Efficacy  2. Safety  3. Immunigenicity | 1. clinical malaria in children during the 18 mo after vaccine dose 3  2. Serious adverse events  3. Anti-circumsporozoite (anti-CS) antibodies | RTS,S/AS01 |
| RTSS Clinical Trial Partnership. [64] | NR | infants  children | N= 6537 | children (age 5–17 months) and young infants (age 6–12 weeks | RCT | block randomisation | 1. Efficacy  2. safety | 1. Diagnosis of severe malaria  2. Serious adverse events | RTS,S/AS01 |
| Sagara et al. [65] | Mali | Adult | N= 120 | Healthy male and female 18–45 years | RCT | block randomisation | 1. Safety and  2.immunogenicity | 1. Adverse event  2. ExoProtein A (EPA) antibody | Pfs25H-EPA |
| Sagara et al. [66] | Mali  Burkina Faso | Children | N= 291 | children aged 5–17 months | RCT | NR | immunogenicity | anti-circumsporozoite antibody response | RTS,S/AS01E |
| Samuels et al. [67] | Ghana  Kenya | Children | N= 1609 | children aged 5–17 months | RCT | web-based randomisation system | 1. Vaccine efficacy  2. Vaccine safety | 1. Episode of clinical malaria  2. Serious adverse event | RTS,S/AS01E |
| Silk et al. [89] | Tanzania | Adults  Children  Infants | 63 | adults (18-35 years), young children (1-6 years) and infants (6-11 months) | double-blind, randomized, controlled trial | NR | safety  immunogenicity | Serious Adverse Events  RH5-specific T cell, B cell and 48 serum antibody | ChAd63-MVA RH5 |
| Sirima et al. [68] | Burkina Faso  Ghana  Uganda  Gabon | Children | N= 1849 | children, aged 12–60 months | RCT | Computer generated block randomisation | 1. Vaccine efficacy  2. Vaccine safety | 1. Episode of clinical malaria  2. Serious Adverse event | GMZ2 |
| Sissoko et al. [69] | Mali | Adults | N= 94 | healthy adults 18–35-year-old | RCT | stratification by village and block randomisation | 1. Vaccine efficacy  2. Vaccine safety | 1. positive thick blood smears and symptomatic malaria  2. Serious Adverse event | PfSPZ |
| Sissoko et al. [70] | Mali | Adults | N= 176 | healthy non-pregnant adults aged 18–50 years | RCT | permuted block randomisation | 1. Vaccine efficacy  2. Vaccine safety | 1. incident malaria infections by thick blood smear  2. Serious Adverse event | PfSPZ |
| Steinhardt et al. [71] | Kenya | Children  Infants | N= 170 | Children and infants (aged 5–9 years, 13–59 months, and 5–12 months) | RCT | permuted block randomization | 1. Safety and Tolerability  2.Immunogenicity | 1. Adverse event  2. Immunoglobulin G antibodies response | PfSPZ |
| Sulaiman et al. [72] | Nigeria | Adults | N= 3377 | Females (1416)  Males (1961) | Cross sectional study | Snow ball sampling | 1. Prevalence  2. determinants  3. Vaccine hesitancy | NR | RTS,S |
| Thera et al. [73] | Mali | Adults | N= 40 | healthy adults aged 18–55 years | RCT | randomization list contained sequential codes linked to vaccine | 1. Safety  2.  immunogenicity | 1. Adverse event  2. anti-AMA1 antibody (IgG) Activity | PfAMA1 |
| Tinto et al. [74] | Tanzania  Burkina Faso | Children | N= 3084 | older children (aged 5–7 years) = 1739  younger children (aged 3–5 years)= 1345 | RCT | computer-generated block randomisation | 1. Malaria incidence  2. Serious adverse event | positive blood film (double or single slide reading) or a positive rapid diagnostic test  fatalities, malaria hospitalization, potential immune-mediated diseases and meningitis | RTS,S/AS01 |
| Tiono et al. [75] | Burkina Faso | Infants    Children | N= 700 | infants and children aged from 5 to 17 months | RCT | NR | 1. Vaccine efficacy  2. Vaccine safety and reactogenicity | 1. Clinical malaria episodes  2. Serious adverse effect | ChAd63 ME-TRAP and  MVA ME-TRAP |
| Umeh et al.[76] | Nigeria | Children | N= 320 | Healthy children aged 5–17 months | RCT | internet-based system randomisation | 1. Vaccine Safety  2.  immunogenicity | 1. Adverse event  2. Assessment of anti-CS surface antigen | RTS,S/AS01 |
| Vera Cruz et al. [77] | Mozambique and Togo | Parents of 0–1-year-old children | N= 227 | Mothers N=120  Fathers  N= 107 | Cross sectional | Cluster sampling | 1. willingness to vaccinate in all circumstances  2. willingness to vaccinate with certain restrictions | NR | NR |
| Witte et al. [78] | Malawi | Infants | N= 480 | infants 1 to 7 days | RCT | standard Statistical Analysis System (SAS) programmed randomization list | 1. Safety  2.Immunogenicity | 1. Adverse events  2. Anti-CS antibody responses | RTS,S/AS01E |

***=studies analysed primary data from existing trials; NR= not reported/unclear, IDI= in-depth interview, FGD= focused group discussion, RCT= Randomized clinical trial, ELISA= enzyme-linked immunosorbent assay, NGO= Non-governmental organizations , NA= Not applicable, +**
